# Antibiotic Prophylaxis in Torso, Maxillofacial, and Skin Traumatic Lesions: A Systematic Review of Recent Evidence

**DOI:** 10.3390/antibiotics11020139

**Published:** 2022-01-21

**Authors:** Enrico Cicuttin, Massimo Sartelli, Emanuele Scozzafava, Dario Tartaglia, Camilla Cremonini, Bruno Brevi, Niccolò Ramacciotti, Serena Musetti, Silvia Strambi, Mauro Podda, Fausto Catena, Massimo Chiarugi, Federico Coccolini

**Affiliations:** 1 General, Emergency and Trauma Surgery, Pisa University Hospital, 56100 Pisa, Italy; enrico.cqtn@gmail.com (E.C.); dario.tartaglia@unipi.it (D.T.); c.cremonini89@gmail.com (C.C.); n_ramacciotti@libero.it (N.R.); s.musetti@hotmail.com (S.M.); sil.strambi@gmail.com (S.S.); massimo.chiarugi@unipi.it (M.C.); 2 Macerata Hospital, 62100 Macerata, Italy; massimosartelli@gmail.com; 3 Unit of Maxillo-Facial Surgery, Pisa University Hospital, 56100 Pisa, Italy; emanuele.scozzafava@ao-pisa.toscana.it (E.S.); bbrevi@gmail.com (B.B.); 4 Department of General and Emergency Surgery, Cagliari University Hospital, 09123 Cagliari, Italy; mauropodda@ymail.com; 5 General and Emergency Surgery Department, Bufalini Hospital, 47521 Cesena, Italy; faustocatena@gmail.com

**Keywords:** antibiotic prophylaxis, trauma, maxillofacial trauma, abdominal trauma, thoracic trauma, burns, skin injury, bites

## Abstract

Use of antibiotic prophylaxis (AP) in trauma patients is a common practice. However, considering the increasing rates of antibiotic resistance, AP use should be questioned and limited only to specific cases. We performed a systematic review of recent literature (from year 2000), aiming to summarize the state of the art on efficacy and appropriateness of AP in patients with traumatic injuries of torso, maxillofacial complex and skin (including burns). Twenty-six articles were selected. In thoracic trauma, AP could be useful in reducing infective complications in tube thoracostomy for penetrating trauma. In maxillo-facial trauma, AP could find a role in the peri-operative trauma setting in the case of a graft or prosthetic implant. In abdominal trauma, there is a lack of consensus on the definition of contamination, infection, antibiotic therapy, and prophylaxis. In burned patients, routine AP is not suggested. In the case of human bites to the extremities, AP could find an indication. Future studies should focus on the subcategories of patients at higher risk of infection, identifying those who would benefit from AP. Attention to antimicrobial stewardship and guidelines focused on AP in trauma are required, to reduce antibiotic abuse, and increase quality research.

## 1. Introduction

Traumatized patients are often characterized by a complex association of lesions, burdened by specific risks of complications. This variety of injuries and severity of scenarios leads to a large heterogeneity of treatments. Furthermore, the complexity of presentation of this category of patients makes standardization and associated clinical research arduous [1]. Antibiotic prophylaxis (AP) is one of the strongest weapons available in preventing infective complications in these patients. However, after decades of liberal use of antibiotics, concerns about the associated risks of such behavior has been raised [2]. With the increasing rates of antibiotic resistance, the concomitant hazard of *Clostridioides* species infection and the urgent necessity to rationalize resources, every physician involved in trauma management should daily question the actual necessity of AP [3]. An ideal AP in trauma should be able to prevent infections, be targeted specifically for the patient and his/her characteristics, cover the risk of infection for a single lesion or association of lesions, reduce the selection of multi-resistant species, and have no adverse effects [4]. However, the chance to operate according to these factors is partially based on the availability of information about the patient at the admission and, in the trauma setting, information is directly related with time. To prevent misuse of AP, we should avoid liberal and premature administration of antimicrobials, preserving AP for specific cases [5]. This can be obtained only based on specific evidence-based indications and relying on antimicrobial stewardship programs [6]. To identify who would benefit from AP in complex trauma scenarios, we performed a systematic review of recent literature to understand the patterns of AP use in clinical practice and to summarize the result of research of the last two decades. We focused on problems directly related to the activity of the general trauma surgeon and the ER physician. Therefore, we analyzed lesions of the face, torso, and skin.

## 2. Methods

**Figure 1:**
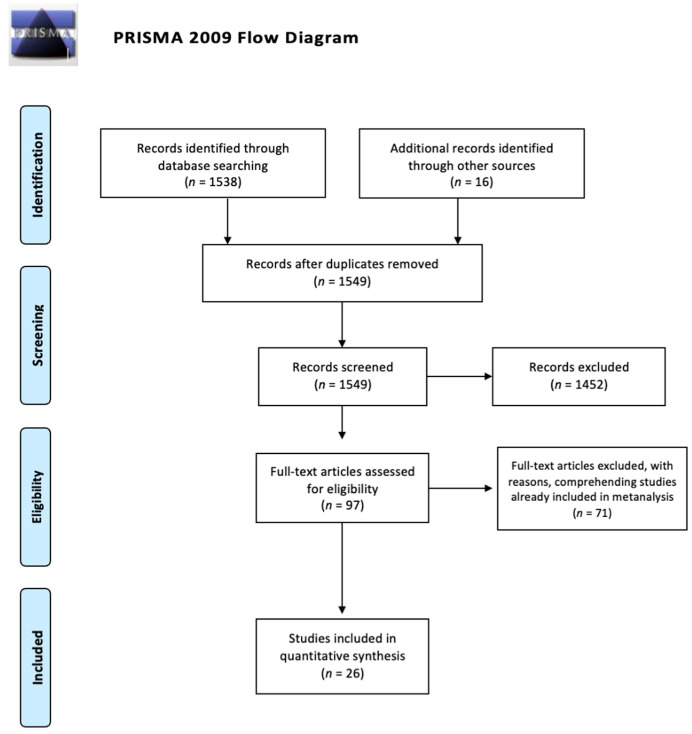
PRISMA flow diagram for research results.

A systematic review of the literature was conducted, based on PRISMA methodology, for all articles on antibiotic prophylaxis in traumatized patients. The reviewers searched in electronic databases (PubMed, Scopus, EMBASE, Cochrane Library) for all articles available in English, published from January 2000 to May 2021. The search terms were “antibiotic prophylaxis”, “trauma”, “antibiotic”, “burns”, “skin”, “maxillo-facial”, “thoracic trauma”, “abdominal trauma”, “facial trauma”, “bites”, and “guidelines”. Combinations of the aforementioned terms were used. The selected articles were screened based on the abstract by the reviewers. Neurosurgical and orthopedic trials were excluded. Expert opinion reviews, narrative reviews, case reports and case series based on less than 30 patients were considered not relevant. Guidelines were included, specifying the grade of recommendation of any statement. If some articles were already included in systematic reviews or meta-analyses, we considered those articles as doubles and we reported the results of reviews and meta-analyses. We searched the bibliography of the selected articles in order to identify additional publications. The reviewers selected articles based on common consensus. The quality of papers was assessed based on the MINORS method for observational studies and the Cochrane collaboration tool for the risk of bias assessment for randomized controlled trials (Table 1) [7,8]. The main outcome was AP efficacy in preventing post-traumatic infections. We considered timing of administration of AP, length of prophylactic treatment, effects of AP on mortality and on hospital length of stay as secondary outcomes. Articles are presented according to a decreasing level of evidence criterium (from systematic review and meta-analysis to retrospective cohort studies). The process is summarized in Figure 1.

## 3. Results

Twenty-six articles were selected. We present our review by dividing the results into five sub-categories: thoracic trauma, abdominal trauma, burns, maxillo-facial trauma, skin wounds (comprehending bites).

### 3.1. Thoracic Trauma

A systematic review and meta-analysis, conducted by Bosman et al. [9] in 2012, investigated the role of antibiotic prophylaxis in tube thoracostomy placement after blunt and penetrating thoracic trauma. The article, based on eleven studies with a total of 1234 patients, showed that antibiotic prophylaxis is useful in reducing the empyema rate (OR 0.32, 95%CI 0.17–0.61). Considering also wound infections and pneumonia, the overall OR for infectious complications was 0.24 (95%CI 0.12–0.49). However, a subsequent subgroup analysis showed that, while the penetrating trauma cohort benefits from antibiotic prophylaxis (OR 0.28 95%CI 0.14–0.57), in blunt trauma patients the effect is not decisive (OR 1.30 95%CI 0.46–3.67).

Heydari et al. [10], in a randomized controlled trial (RCT) from 2014 of 104 cases of tube thoracostomy after blunt trauma, showed no significant effect of the prophylaxis in preventing empyema and pneumonia.

A prospective study by Bradley et al. [11] considered the role of retained hemothorax in the development of infectious complications after thoracic trauma. In this study, 328 patients with retained hemothorax were analyzed. The failure to administer peri-procedural antibiotics on thoracostomy tube placement was recognized as an independent predictor of pneumonia (OR 2.6 95%CI 1.3–5.4), along with ISS (Injury Severity Score) > 25 and blunt mechanism of trauma.

A second multicenter prospective observational study by Cook et al. [12], based on 1887 patients who underwent tube thoracostomy placement after traumatic hemopneumothorax, demonstrated that no difference can be found between the antibiotic vs. the non-antibiotic group in regards to infection incidence (2.2% vs. 1.5% respectively, *p* = 0.75). Antibiotic use was positively but non-significantly associated with risk of pneumonia (OR 1.61; 95%CI 0.86–3.03; *p* = 0.14) and empyema (OR 1.51; 95%CI 0.42–5.42; *p* = 0.53).

A retrospective study, based on 1002 patients and published by Kong et al. [13], investigated the role of antibiotic prophylaxis in tube thoracostomy after trauma in a developing world setting. The cohort was composed of both penetrating and blunt trauma. The difference in incidence of empyema in the two groups was not statistically significant. The patients who developed empyema had hemothorax as a diagnosis; no patient with pneumothorax developed infective complications. The authors stated that, considering the low incidence of post traumatic empyema (1.5%, 95%CI 0.8–2.5%), the routine use of antibiotic prophylaxis for tube thoracostomy in post-traumatic thoracic injuries is not justified in a developing world setting.

The results are summarized in Table 2.

### 3.2. Abdominal Trauma

Abdominal trauma, due to its intrinsic high risk of contamination, lays between the possibility of prophylaxis and the necessity of therapy.

The difficulty to obtain high quality data is shown by three subsequent attempts to review RCTs about the usefulness of prophylaxis in abdominal penetrating trauma: all of them failed because of lack of adequate studies and, therefore, are not included in the review [14,15,16].

Nonetheless, some authors proposed guidelines to standardize the therapeutical behavior in case of abdominal trauma. In 2012, the Eastern Association for the Surgery of Trauma (EAST) [17] proposed a guideline for the management of antibiotic prophylaxis in penetrating abdominal trauma. The authors stated as Level I recommendation (based on high level of evidence, according to the methodology established by the Agency for Health Care Policy and Research of the US Department of Health and Human Services and Oxman indications [18]) that antibiotic prophylaxis should be administered only for 24 h in the presence of hollow viscus injury and that no data support the extension of prophylaxis over 24 h in the case of damage-control laparotomy. Concerning the choice of antibiotic, the authors stated that a broad spectrum one, with coverage of both aerobic and anaerobic bacteria should be preferred (Level I) and that aminoglycosides should be avoided (Level III). Further attention was given to the necessity to increase the antibiotic dosage in case of hemorrhagic shock (Level III). However, we consider hollow viscus damage with contamination to be an indication for antibiotic therapy, more so than for prophylaxis.

In 2019, the Korean Society of Acute Care Surgery published a guideline [19] about the use of antibiotics in patients with abdominal injuries. The authors stated that patients not requiring surgery do not need antibiotics (Level of recommendation 1A, according to GRADE recommendations [20], based on RCTs). However, the statement, graded with the strongest of recommendations, was indirectly obtained by considering the administration of antibiotics in the case of penetrating trauma as the gold standard. In fact, there are no comparative blind studies on whether to administer antibiotics or not in penetrating trauma. The ethical problems related to high expected morbidity and mortality in a control group (receiving no antibiotics) make the study design on this topic critical.

Goldberg at al. [21] investigated the role of antibiotics in damage control laparotomy (DCL). A retrospective review of 121 patients was performed. The study showed that post-operative antibiotic administration (OR 6.7% 95%CI 1.33–33.8, *p* = 0.044) and bowel injuries (OR 3.45, 95%CI 1.03–11.5, *p* = 0.02) were positive predictors of infection, while pre-operative administration of antibiotics was a negative predictor of infection (OR 0.20 95%CI 0.05–0.91, *p* = 0.037). On the other side, neither ISS nor DCL were independent predictors of infection. However, the study did not discriminate between therapy and prophylaxis, and the heterogeneity of lesions investigated makes it difficult to obtain a proper indication about AP.

A retrospective comparative study published by Smith et al. [22] explored the differences between two groups of trauma patients, both penetrating and blunt, who underwent trauma laparotomy. The first group (151 patients) followed the prophylaxis guidelines by Surgical Care Improvement Project (SCIP) [23], the second one (155 patients) was not compliant to the indications. The two groups were adjusted for ISS, hypotension, blood transfusion, enteric injury, operative duration and other confounding factors. The group treated according to the SCIP guidelines had an inferior risk of developing SSI (OR 0.43, 95%CI 0.2–0.94, *p* = 0.035), compared to the other group. The authors stated that SCIP guidelines could be useful in a trauma setting, even in elective surgery and if mortality in the two groups was not affected (*p* > 0.05) by adherence to the guidelines. However, it is not clear which patients received prophylaxis alone and which received antibiotic therapy. In fact, one of the differences between the two groups was the average duration of in-hospital antibiotic therapy (4 vs. 9 days, *p* < 0.001).

The duration of AP (>24 h or ≤24 h) and the choice of antibiotic in a penetrating abdominal trauma setting have been investigated by a recent systematic review of RCTs [24]. Of 29 RCTs, 23 were deemed to be at high risk of bias. The largest part of these studies was conducted more than 20 years ago. The results were not definitive: the authors were uncertain about the benefit of AP longer than 24 h on abdominal SSI (RR 1.00, 95%CI 0.81 to 1.23; I^2^ = 0%; 7 studies, 1261 participants; very low-quality evidence), mortality (Peto OR 1.67, 95%CI 0.73 to 3.82; I^2^ = 8%; 7 studies, 1261 participants; very low-quality evidence), or intra-abdominal infection (RR 1.23, 95%CI 0.84 to 1.80; I^2^ = 0%; 6 studies, 111 participants; very-low quality evidence). In regards to the choice of the appropriate drug, based on 2020 patients, the low quality of evidence did not allow the authors to give further suggestions about the best prophylactic regimen.

The results are summarized in Table 3.

### 3.3. Maxillofacial Trauma

In 2006, Andreasen et al. [25] published a systematic review about prophylaxis in maxillofacial fractures, selecting four RCTs. The authors concluded the study recognizing an effect of antibiotic administration in reducing the infection rate 3-fold. Moreover, 1-shot regimens or short protocols (max one day) seemed to have an equal if not better effect, compared to longer administrations, especially considering open reductions. The statement was limited to fractures not involving the condylar region, as the trials included fractures related to the dental area of the mandible. Generally speaking, no difference in effect was found, neither among different classes of antibiotics, nor according to the location of the fracture (condylar, maxillary, or zygoma).

In 2018, Habib et al. [26] published a systematic review and meta-analysis, focusing on suitability of post-operative prolongation of AP. Based on 13 trials (7 RCTs and 6 cohort studies), the authors stated that addition of post-operative antibiotics to the standard perioperative prophylaxis does not significantly decrease the likelihood of SSI (Surgical site infection) in the patient undergoing surgery for maxillofacial trauma (RR 1.11 95% CI 0.86–1.44). The result was confirmed even in sub-analysis: avoiding post-operative antibiotic administration does not increase the infection rate in mandibular fractures (eight studies, RR 1.02 95% CI 0.62–1.67) or when open reduction is needed (seven studies, RR 1.00 95% CI 0.61–1.67). The result did not differ significantly after sensitivity analysis. Restriction to RCTs gave similar results (RR 1.00 95% CI 0.62–1.67) and no significant difference with cohort studies (RR 1.21 95% CI 0.89–1.63) was found.

In 2020, Delaplain et al. [27] performed a systematic review and meta-analysis to demonstrate the efficacy of prolonged AP in reducing SSI after any type of facial fracture. Twenty-seven studies were selected. Of these, 16 studies focused on mandible fractures, four studies on mid-face fractures, and six studies on orbital fractures. Pooled analysis for the rate of infection in different types of fractures showed no significant difference. In mandible fractures, <24 h AP was compared to 24–72 h AP and >72 h AP: calculated ORs did not favor prolonged prophylaxis. In general, no significant effect on reducing infection rate with prolonged AP was found by the authors.

In 2021, Dawoud et al. [28] published a systematic review and meta-analysis, including 7 RCTs and 9 retrospective studies. The authors stated that, when comparing patients who received AP with those who received none, there was no clear advantage of AP in reducing adverse effects. However, there was high heterogeneity (*p* = 0.02, I = 69%), with the confidence interval crossing the line of no effect of the intervention (RR: 1.38, 95% CI: 0.47–4.03). Prolonged (>1 day) AP did not show any benefit (RR 0.84; 95% CI: 0.54–1.31), and neither did preoperative vs. postoperative administration of AP (RR 1.47; 95% CI 0.74–2.89). In regards to IV and oral administration, both in preoperative and postoperative settings, no clear benefit was found.

The recent guidelines by the Surgical Infection Society (2020) [29] regulate the use of antibiotics in facial fractures, focusing on pre-operative, peri-operative and post-operative periods. With attention towards the type of fracture, five different scenarios and five indications were given (GRADE method [20]): avoid antibiotic prophylaxis in non-operative facial fractures (Level 2C); avoid prescribing prophylaxis in operative non-mandibular fractures during the pre-operative period (Level 2C); avoid prescribing pre-operative prophylaxis in operative mandibular fractures (Level 2C); avoid antibiotic prescriptions in the post-operative period (>24 h) for non-mandibular fractures (Level 1B); avoid antibiotic prescription in the post-operative period (>24 h) for mandibular fractures (Level 1B). The results are summarized in Table 4.

### 3.4. Burns

Avni et al. [30], in 2010, published a meta-analysis based on 17 studies about AP (12 systemic prophylaxis, 5 topical). The authors stated that systemic prophylaxis reduced all-cause mortality (OR 0.54; 95%CI 0.34–0.87), according to 5 studies with no heterogeneity (*p* = 0.21). The systemic prophylaxis was also related to a reduction in the rate of pneumonia (OR 0.55; 95%CI 0.36–0.84). On the counterpart, based on 4 trials, prophylaxis administered pre-operatively (3 trials) and non-absorbable antibiotics (1 trial) did not affect mortality. Concerning wound infection, four trials showed a borderline positive effect of perioperative prophylaxis (OR 0.72; 95%CI 0.52–1.01). Bacteriemia was not affected by any intervention. Higher efficacy of prophylaxis in reducing Gram+ infections was highlighted (OR 0.58; 95%CI 0.43–0.76), but the same efficacy was not reported for Gram− infections. Limitations of the study were represented by the large chronological span covered by the considered trials and by the general low quality of evidence.

In 2013, Barajas-Nava et al. [31] conducted a systematic review and meta-analysis about the role of AP in preventing burn wound infection. The review comprised 36 RCTs, with a cumulative population of 2117 patients. Twenty-six studies analyzed the use of topical antibiotics, seven analyzed systemic antibiotics, two analyzed non-absorbable antibiotic regimens, and one study analyzed local antibiotics administered via the airway. Additionally, the authors performed a sub-analysis of 11 studies focused on the use of topical silver sulfadiazine. In the topical antibiotic studies group, two trials (99 patients) compared neomycin, bacitracin, and polymyxin B with inactive control: in patients with wound infection, no significant difference between treatment group and control group (OR = 0.75; 95%CI 0.32–1.73) was found. No significative difference was found related to sepsis, antibiotic resistance, wound healing, hospital length of stay or infectious-related mortality, too. However, all of the studies were considered to have a high risk of bias. Regarding the use of silver sulfadiazine, the authors highlighted a significant increase in wound infection compared with dressing or skin substitute (OR 1.87; 95%CI 1.09–3.19) and in total length of hospital stay (MD 2.11 days; 95%CI 1.93–2.28). Again, the studies were at a high risk of bias. Concerning the systemic administration of antibiotics, after pooled analysis and removal of meta-analysis with high heterogeneity, no definitive benefit in using antibiotic prophylaxis was found. However, trimethoprim-sulfamethoxazole alone was associated with a significant decrease in the risk of pneumonia, according to one trial (RR 0.18; 95% CI 0.05–0.72). Regarding perioperative systemic prophylaxis, there was no effect on infection rate or any other outcome of the review (sepsis, mortality, hospital length of stay, wound healing). Use of non-absorbable antibiotics was related to an increased rate of MRSA when associated with cefotaxime (RR 2.22; 95% CI 1.21–4.07), with no effect on general rate of infections. Moreover, airway-administered antibiotics had no effect on sepsis or mortality compared with placebo. In conclusion, considering the evidence available and the strong limitations of analyzed trials, the review was not able to determinate strong evidence supporting AP in burn wound infection.

In 2019, Csenkey et al. [32] published a meta-analysis about the efficacy of systemic AP in pediatric burn injury. Including 6 studies and 1735 pediatric patients, the authors were unable to find evidence supporting the routine use of antibiotic prophylaxis. The risk of developing an infection, local or systemic, was not different in the treatment group when compared to the no-antibiotic group (OR 1.35; 95%CI 0.44–4.18). The same result applies to the two categories when systemic complications alone were considered (OR 0.74; 95%CI 0.38–1.45). The authors performed a sub-analysis that included burned surface area, age, country, and income level. However, they failed to demonstrate any benefit of AP.

In 2017, Ramos et al. [33] conducted a systematic review about the use of systemic AP in burn patients, both pediatric and adult. The paper considered 19 trials as suitable for further analysis. The results were divided into systemic prophylaxis in early post-burn patients, with severe and non-severe lesions, and in patients undergoing surgery. Of the 13 studies regarding early post-burn patients, six analyzed non severe burns (less than 20% of total body surface area), while the remaining considered the antimicrobial prophylaxis in severe burns. The author used the GRADE system to summarize the results of the review [20]. In conclusion, in the early post-burn period, AP has no indication in most burn patients (GRADE 1C) but it could find an indication in mechanical ventilated patients with severe burns (GRADE 2B). At the same time, perioperative prophylaxis could be useful for the prevention of split-thickness skin graft infection in selected procedures (Grade 2B), while, during resection of devitalized tissues, the prophylaxis did not find a proper indication (GRADE 2B). However, the trials were recognized at risk of bias: the time span was wide (1982–2016), some studies were of poor quality, and there was no homogeneity in type of drugs, doses, and duration of treatment.

Muthukumar et al. [34], in 2019, published a retrospective study based on 157 patients. Seventy-seven patients received AP, the remaining 80 patients did not. There was no significative difference in mortality or sepsis rate. However, a sub-analysis focused on patients with inhalation burns and showed a significative difference in mortality between those who developed pneumonia in the prophylaxis group and those who developed pneumonia in the non-prophylaxis group. The authors recognized prophylaxis as potentially useful in burn patients with airway involvement and subsequent risk of pneumonia.

Yeong et al. [35], in 2020, focused the attention of their study on the characteristics of wound microbiology and on the outcomes following systemic antibiotic prophylaxis in mass burn casualties. Even with the strong limitation of a small patient sample (31 patients), the study showed that multidrug-resistant organisms were found in 39% of the patients one month after admission. Patients were treated with systemic AP from 2 to 14 days, according to an arbitrary estimation of infection probability. This risk of developing multi-resistant pathogens was extremely high in patients with more than 40% of the body involved (OR 41.7; 95%CI 2.1–810.7 *p* = 0.01) and in those who received two or more different classes of antibiotics (OR 9.9; 95%CI 1–92.7 *p* = 0.04). However, even considering the data appealing, the wide confidence intervals suggest that care should be taken with these results. In conclusion, a strong limitation of this study is represented by the small and heterogenous cohort included.

The results are summarized in Table 5.

### 3.5. Skin Wounds and Bites

Lesions of soft tissues occupy an important spot in ED activities. The necessity of prophylaxis, often suggested and applied by physicians, needs to be assessed. Most randomized trials on the topic were published before the year 2000 and they are not directly included in this specific review.

Specifically speaking about traumatic lesions of the skin, some narrative reviews appeared during the last years. However, for methodological reasons and because these articles are based on trials conducted before year 2000, they were not considered in this review.

In 2009, Gerhardt et al. [36] published a retrospective cohort study (53 patients) about the usefulness of wound irrigation and systemic antibiotic prophylaxis (SAP) in mild combat injuries. The rate of infection was 17% in the irrigation/no SAP group, 40% in the SAP/no irrigation group, and 75% in the no SAP/no irrigation group (*p* < 0.0005). The authors hypothesized a synergistic mechanism of action of the two prophylactic treatments and confirmed the adequacy of irrigation in complex wounds at risk of infection.

Lloyd et al., in 2018 [37], considered the effect of antibiotic prophylaxis in combat-related open soft tissues wounds in a retrospective study based on 287 patients. The study compared infectious outcomes in narrow prophylaxis regimens (as indicated by Department of Defense guidelines) and expanded gram negative coverage. No significant difference was found between the two groups in terms of infection rate (*p* = 0.345) and length of hospitalization. The trial did not consider the possibility of avoiding prophylaxis.

A third trial on combat injuries, published by Weintrob et al. [38] in 2018, prospectively analyzed the injuries of 1807 patients, reporting an early infection rate of 34%. Half of the infections affected skin, soft tissue or bones. Amputation, transfusions, severity of injury, need for mechanical ventilation, and intensive care unit admission were related to risk of infection, while antibiotic administration and early operation were not.

Concerning infection risk in bites, in 2001 (edited with no changes to conclusions in 2008), Medeiros and Saconato [39] published a systematic review and meta-analysis about the suitability of AP after mammalian bites. Eight studies were included (two pediatric, two only adults, three mixed, one without specification). All studies analyzed the incidence of infection of the wound after the bite, while only four studies considered the different incidence according to the location of the wound. Three studies considered the type of wound (puncture, laceration, or avulsion). Six studies focused on dog bites, one on cats, and one on humans. Overall, there was no significant benefit in using AP for mammal bites (OR 0.49 95% CI 0.15.1.58). However, after using a fixed effect model to reduce heterogeneity, the authors showed an apparent weak benefit in using AP (OR 0.39, 95% CI 0.19–0.77). AP was considered effective in reducing infectious complications in human bites (OR 0.02, 95% CI 0–0.33), but no clear benefit was found for dog and cat bites. However, human and cat bites were studied only in one trial, respectively. While the type of wound seemed to not influence the rate of infection, injuries located at the hand showed a higher rate of complication if not treated with antibiotic prophylaxis (2% in antibiotic group vs. 28% in control, OR 0.1 95% CI 0.01–0.86).

In 2009, Looke and Dendle [40] published a systematic review on prophylaxis and treatment in mammal bites. Considering the review previously described [39] and a subsequent RCT about human bites [41], the authors stated that prophylaxis did not add any benefit even in human bites, when considering low risk areas (not feet, hand and over cartilaginous areas). Yet, concerns about the quality of studies were raised.

The results are summarized in Table 6.

## 4. Discussion

### 4.1. Thoracic Trauma

Contamination of the thoracic cavity and infective complications of thoracic trauma are a major concern. Mortality for thoracic trauma prior to the introduction of antibiotic therapy was around 60%, decreasing during the Korean war to 2% [42] when antibiotics were widely used. After thoracic trauma, from 70% to 90% of patients will need tube thoracostomy [9]. Therefore, focusing on risks of infection in patients needing invasive maneuvers, the scientific community has investigated methods to reduce infection rate. Retained hemothorax (RH) is recognized as a risk factor for developing pneumonia and empyema [11], alongside with pathological contact with the outside environment in penetrating trauma [43]. Tube thoracostomy can solve both pneumothorax and hemothorax, evacuating the content of the thoracic cavity and reducing the incidence of subsequent empyema. Nevertheless, the post-traumatic empyema rate varies from 2% to 25%, with *S. aureus* responsible for 35–75% of subsequent infections [9]. For these reasons, the usefulness of unconditioned AP in tube thoracostomy has been evaluated. The literature is quite concordant in considering that the advantage of AP in tube thoracostomy placement for blunt trauma is nonexistent [9,10], even if some studies showed a higher rate of pneumonia in blunt trauma [43]. One prospective study showed a different tendency in patients with RH [11], identifying blunt trauma as an independent predictor for post-traumatic pneumonia, alongside failure to administer AP. However, the study is based only on patients with RH, which is a risk factor for infection. This underlines the necessity to assess the risk for infection based on a thorough evaluation of patient-related and trauma-related risk factors, together with the ability to recognize precociously eventual signs of infection. In penetrating trauma, there is no such strong agreement on avoiding AP [9,43]. The source of contamination in penetrating trauma, leading to higher risk of pneumonia and wound infection, has been investigated. Some authors theorized a role of tube placement itself in contamination of the wound with skin microbiota [43], with the same mechanism of penetrating trauma. In the same fashion, pre-hospital tube placement was considered controversial, because of a theoretical increased risk of contamination. However, the infection rate does not differ between pre-hospital and in-hospital tube placement [44]. Again, the factors involved in infective risk assessment in thoracic trauma are various and complex. This is well explained by the heterogeneity of patients’ characteristics involved in the studies considered by this review and by the general low-medium quality of the trials. In conclusion, further randomized controlled trials are needed to investigate the role of AP, with particular attention to sub-categories of traumatized patients with different general risks of infection and different types and severity of thoracic lesions.

### 4.2. Abdominal Trauma

Abdominal trauma is characterized by a large variety of different presentations and by high risk of contamination of the peritoneal cavity. This risk is maximal when a penetrating mechanism is involved. Before the antibiotic era, mortality for colonic penetrating trauma accounted for 60–70% of cases [42]. Historically speaking, some attempts have been made to stratify risk for intrabdominal infection following penetrating trauma of the abdomen, according to the time of antibiotic administration [45]. In the same fashion, a trial investigated the best type of antibiotic prophylaxis, underscoring the necessity of anaerobic coverage [46]. After these seminal trials, confronting antibiotic treatment vs. placebo was considered unethical. According to more recent guidelines actually available on the topic, AP is usually not suggested when there is no necessity of surgery [19] and, in patients with penetrating trauma undergoing surgery, should not be continued over 24 h even when hollow viscus injury is present [17]. Unfortunately, data quality is strongly biased by the complexity of randomization in an emergency setting [47] and by the lack of correct division of patients in four different cohorts: those not receiving any AP, those receiving proper AP, those receiving short antibiotic therapy after peritoneal contamination, and those needing prolonged therapy for intra-abdominal infection. Even when focusing on duration of prophylaxis and type of antibiotic, no definitive evidence suggesting a precise clinical behavior was found [24]. To overcome this general deficiency of strong evidence, the tendency to use guidelines meant for elective settings in trauma settings became common, and this could have had a positive outcome [22]. However, this behavior could lead to the wrong consideration of a physio-pathological similarity between the elective and the trauma patient. This review highlights the need for a strong consensus on defining the degree of peritoneal contamination, intrabdominal infection, prophylaxis and therapy in an abdominal trauma setting. Starting from this point, it will be possible to create studies with a lower grade of bias. Concerning AP duration, Herrod and colleagues [24] failed to demonstrate a clear advantage in prolonging AP over 24 h in penetrating abdominal trauma. In addition, no antimicrobial was found to be superior to another. All the RCTs included were published before the year 2000.

### 4.3. Maxillo-Facial Trauma

Infection is the most common complication reported with open mandibular fractures in facial trauma (10–15%) [48]. With the advent of perioperative antibiotic therapy and plate fixation, infection rates have been reduced compared with the wire osteosynthesis era, but they remain a challenge even for experienced surgeons. Most studies focused on the mandible area. Upper face (frontal region) and mid face (naso-ethmoidal region) are often studied together since they share the sinus cavities. General risk of contamination is due to the colonization of oral cavity with the bacterial microbiome. Therefore, fractures involving this site could be considered contaminated [49]. However, not all the fractures will develop infection. AP plays an undoubtful role in reducing the rate of infection [25], but a strong need for regulation emerged. In 2015, Brooke [50] published the results of a survey conducted among 205 surgeons (maxillo-facial, plastic, and otolaryngologist), showing a wide lack of consensus in AP administration for operative and non-operative facial fractures. There was no uniformity in duration of AP or in the choice of antibiotic. Recent evidence, summarized by Habib in a meta-analysis [26], focused on the necessity to reduce the use of AP in trauma related fractures of the face, after decades of liberal practice. Relying on a standard peri-operative antibiotic administration, the meta-analysis demonstrated that less than 24 h of AP are more than sufficient to guarantee the best effect on infection rate [26]. The same result was empowered by Delaplain’s results [27], who stated that anatomical location of the fracture, if important for surgical planning and strategy, does not influence the rate of infection rate when a short course of AP is prescribed. Considering mandible fractures, Dawoud [28] confirmed these findings with the latest meta-analysis published on the topic. However, the authors stated a high risk of bias of the studies considered, with a clear weakening of the results and a capital need for stronger evidence. The latest guidelines by SIS [29] further reduced the role of AP in maxillo-facial trauma, limiting use of short antibiotic administration to cases needing surgery intervention and setting a new direction in considering the appropriateness of AP.

### 4.4. Burns

In burned patients, infection is a capital concern, frequently being the cause of death or skin graft loss [51]. Considering the tremendous effect of multi-resistant species in these patients, the usefulness of AP, both systemic and topical, has been evaluated by several studies. While Avni [30] stated that a reduction in all-cause mortality was related to systemic AP, other authors [31,32,33,34] did not claim a definitive benefit in this practice. Avni’s result could be explained by the secondary effect of prophylaxis in reducing secondary complications (i.e., in this study, pneumonia): the authors illustrated that the reduction in mortality was obtained by excluding the effect of perioperative AP from the analysis. All of this came at the cost of an increased bacterial resistance and antibiotic abuse was strongly suspected as a response of Avni’s study [52]. However, when focusing on the ability of AP to reduce the burn infection rate, all trials analyzed here were quite concordant in considering that the routine administration of AP was not definitively useful, both in adult and pediatric populations. The Cochrane review by Barajas-Nava [31] was able to rule out the inappropriateness of silver sulfaziadine in burn treatment, showing that better results are obtainable with dressing and skin substitutes. This statement brings value to the primary role that adequate source control (here represented by protection on the lesioned area and removal of contaminated material) has in reducing the infective risk. Other systemic attempts to reduce infections, including non-absorbable antibiotics, failed to demonstrate their value or, in the worst scenario, increased the risk of multi-resistant bacteria onset [31]. We should concentrate on selecting those who would benefit from AP. As highlighted by Ramos [33], AP could protect the most fragile patients from complications. Therefore, a possible role for AP was suggested in mechanically ventilated patients with severe burns, in patients with airway involvement and, perioperatively, to prevent graft infection [33,34]. To add fundamental contributions to this topic, further RCTs focusing on specific classes of risk among these patients are needed. Moreover, strong awareness of the risk of creating antimicrobial resistance inside a burn unit should always be present. Additional specific research on alteration of pharmacokinetics in burned and severe trauma victims could give more sharpness to any indication coming from results of specific populations [53,54].

### 4.5. Skin Wounds and Bites

According to US data, almost 12 million skin wounds are treated every year in American Emergency departments, adding to the count another 1.5 million bites [55]. Even taking into account the enormous impact of this issue on the community, the high-quality literature (RCTs and meta-analyses) on skin wounds is based on evidence older than twenty years. To avoid AP in non-complicated skin wounds can be considered a well-established practice [56], but the absence of clear and recent guidelines could lead to permissive prescription of antibiotics. Since the numbers aforementioned, even a small percentage involved in this relative lack of regulation could bring major consequences in terms of costs, adverse effects, and antibiotic resistance. In regards to cohort studies, the experience coming from the military setting could weaken and reduce the usability of findings in civilian situations [57]. In regards to bites, the literature highlights the necessity to take into consideration the general conditions of the patients and the characteristics of the bite [39]. Recent evidence contributed to the importance of location in considering AP [40] in the case of mammalian and human bites.

## 5. Conclusions

Nowadays, the utility and appropriateness of AP in the trauma setting is questioned. Literature on the topic is often dated to the years of large diffusion of new antimicrobials. In more recent years, the reduced number of novel molecules and a decreased concern of infective complications has shifted the attention towards other topics. At the same time, a permissive use of antibiotics has caused increased antimicrobial resistance and added urgency to the issue. A change in mindset is essential; a new awareness is required, and research should follow the necessities of indications required by the community. This review showed that, in most situations, AP does not give any major advantage in reducing infection rate in the trauma setting. Attention to general conditions of the patients and factors impairing the immunity response should be given. In thoracic injuries, AP could reduce the infection rate in tube thoracostomy for penetrating trauma. Considering maxillo-facial trauma, AP could find a role in the peri-operative trauma setting, especially when graft or prosthetic implant is involved. However, no definitive benefit of any specific regimen of AP can be assessed. Long courses of AP do not add any advantage in reducing the infective risk. In regards to abdominal trauma, no clear statement about AP can be given and a consensus on definition of contamination, infection, antibiotic therapy, and prophylaxis is needed. In burned patients, routine AP is not suggested. Specific infection risk of the patient should be considered, with source control playing a major role in prevention of infection. Concerning skin trauma, AP should be considered after human bites in high risk areas (hand, extremities). Limitations of this study include the high heterogeneity of the studies included (thoracic trauma, maxillo-facial trauma, burns), the lack of recent and homogeneous evidence (skin lesions), and the absence of evidence and consensus on definitions (abdominal trauma). Attention to antimicrobial stewardship and guidelines focused on AP in trauma are required, in order to reduce antibiotic abuse, increase data coherence and facilitate homogeneous, high-quality research.

## Figures and Tables

**Table 1:** Quality assessment of included trials.

**Non-Randomized**		**Quality Evaluation Criteria**								**Additional Criteria in Comparative Studies**				
Study (Ref.) Year		Clear Stated Aim	Inclusion of Consecutive Patients	Prospective Data Collection	Endpoints Appropriate to the Study Aim	Unbiased Assessment of Study Endpoint	Appropriate Follow-Up Period	Loss to Follow-Up Less Than 5%	Prospective Calculation of the Study Size	Adequate Control Group	Contemporary Groups	Baseline Equivalence	Adequate Statistical Analysis	Total
1	Bradley 2013	2	2	2	2	2	2	2	0	2	2	2	2	22/24
2	Cook 2019	2	2	2	2	2	2	2	0	2	2	2	2	22/24
3	Kong 2015	2	2	0	2	2	2	2	0	-	-	-	-	12/16
4	Smith 2021	2	2	0	1	2	2	2	0	2	2	2	2	19/24
5	Goldberg 2016	2	2	0	1	2	2	2	0	2	2	1	2	18/24
6	Muthukumar 2019	2	2	0	1	2	2	2	0	2	2	2	2	19/24
7	Yeong 2020	2	1	0	1	2	2	1	0	-	-	-	-	9/16
8	Gerhardt 2009	2	1	0	2	2	1	1	0	1	2	1	2	15/24
9	Lloyd 2018	2	2	0	2	2	2	2	0	2	2	2	2	20/24
10	Weintrob 2018	2	2	2	2	2	2	2	0	-	-	-	-	14/16
**RCT**		**Quality Evaluation Criteria**												
Study (Ref.) Year		Randomization		Allocation Concealment		Blinding of Partecipansand Personnel		Blinding of Outcome Assessment		Incomplete Outcome Data		Selective Reporting	Other Bias	
Heydari 2014		?		?		?		?		+		+	?	

For non-randomized trials items are scored as follows: 0 (not reported), 1 (reported but inadequate), 2 (reported and adequate). Global ideal score for non-comparative studies is 16 and for comparative ones is 24. For randomized controlled trials (RCTs): Low risk of bias: +; High risk of bias: -; Unclear risk of bias: ?

**Table 2:** Summary of studies included on antibiotic prophylaxis in thoracic trauma (AP: antibiotic prophylaxis; RCT: randomized controlled trial; ISS: Injury Severity Score).

Author	Year	Study Type	Intervention	N. of Patients	Result	Limitations
Bosman	2012	Systematic review and meta-analysis of RCTs	Infection rate in tube thoracostomy (AP vs. noAP)	1234	Reduced empyema rate in AP group; reduced risk of infection in penetrating chest trauma; no effect on blunt trauma	Wide time span of trials (1977–2009); no consensus on definition of infective complications; no indications on length of AP.
Heydari	2014	RCT	Infection after thoracostomy in blunt trauma (AP vs. noAP)	104	No reduction in infection rate from 24 h AP after tube thoracostomy for blunt trauma	Small sample; limited to blunt trauma
Bradley	2013	Prospective	Risk factors for pneumonia in patients with post-traumatic retained hemothorax	328	ISS > 25, blunt trauma, and failure to administer peri-procedural antibiotic are independent predictors of pneumonia	Observational study, limited to patients with retained hemothorax
Cook	2019	Prospective	Pneumonia and empyema after tube thoracostomy (antibiotic vs. no-antibiotic)	1887	No difference of incidence in the two groups; no significative association of AP with infection risk	Observational study, no clear division of AP and antibiotic treatment
Kong	2015	Retrospective	AP vs. noAP after tube thoracostomy in developing setting	1002	No difference in empyema rate in the AP vs. noAP.	Observational study, no indication about other types of infective complications

**Table 3:** Summary of studies included on antibiotic prophylaxis in abdominal trauma (AP: antibiotic prophylaxis; AT: antibiotic therapy; RCT: randomized controlled trial; SSI: surgical site infection; SCIP: Surgical Care Improvement Project; EAST: Eastern Association for the Surgery of Trauma; KSACS: Koreas Society of Acute Care Surgery).

Author	Year	Study Type	Intervention	N. of Patients	Result	Limitations
Goldberg (EAST)	2012	Guidelines (review)	AP in penetrating abdominal trauma	-	AP only for 24 h in presence of hollow viscus injury; broad spectrum antibiotic (anaerobic and aerobic coverage); increase AP dosage in blood loss.	Hollow viscus injury and contamination of peritoneum can be considered indication for AT; limited only to penetrating trauma
Jang (KSACS)	2019	Guidelines (review)	Use of antibiotic in patients with abdominal injuries	-	Indication: if no surgery is needed, no AP is needed.	Statement indirectly obtained, no recent RCTs.
Smith	2012	Retrospective	Effect of SCIP guidelines on abdominal trauma patients (adherence to guidelines vs. no adherence)	306	Group treated according to SCIP guidelines had inferior rate of SSI; no differences in mortality	No clear distinction between AP and AT
Goldberg	2016	Retrospective	Use of antibiotics in damage control laparotomy	121	Pre-operative antibiotic reduces infection rate; post-operative antibiotic and bowel injury increases infection risk.	No distinction between AP and AT; heterogeneity of lesions and scenarios
Herrod	2019	Systematic review and meta-analysis of RCTs	Choice and duration (<24 h vs. >24 h) of AP in penetrating abdominal trauma	4458	No definitive indications. Uncertainty on specific regimen and duration of AP.	All studies published more than 20 years ago; 23/29 studies at high risk of bias; limited to penetrating trauma.

**Table 4:** Summary of studies included on antibiotic prophylaxis in maxillo-facial trauma (AP: antibiotic prophylaxis; RCT: randomized controlled trial; SSI: surgical site infection; IV: intravenous; SIS: Surgical infection Society).

Author	Year	Study Type	Intervention	N. of Patients	Result	Limitations
Andreasen	2006	Systematic review of RCTs	Efficacy of AP in reducing infection rate; duration of AP (One-shot and <24 h vs. >24 h)	573	3-fold reduction of infection rate in AP group; one-shot and 24 h AP similar or better than >24 h AP; no differences based on the facial region involved	Wide time span of studies included (1975–2001); quasi-randomized studies included
Habib	2018	Systematic review and meta-aanalysis of RCTs and cohort studies	Efficacy of addiction of post-operative AP in reducing infection rate vs. pre-operative/peri-operative AP alone	2236 (635 RCTs + 1601 cohort studies)	No reduction in infection rate when adding post-operative prophylaxis.	Lack of sub-analysis referred to patients at high risk for infection
Delaplain	2020	Systematic review and meta-analysis of RCTs and cohort studies	<24 h AP, 24–72 h AP, >72 h AP comparison in reducing SSI rate; risk according to fracture location	3132 (2316 mandible + 377 orbital + 439 mid-face)	Prolonged AP (>24 h) does not reduce SSI risk; no differences of SSI among different location of fracture; AP > 72 h could increase SSI rate of mandible fractures	Lack of sub-analysis referred to patients at high risk for infection
Dawoud	2021	Systematic review and meta-analysis of RCTs and cohort studies	Efficacy of AP in reducing infection rate in mandibular fracture (AP vs. noAP; short AP vs. long AP; preop. AP vs. preop. + postop. AP; preop. And postop. IV + oral AP vs. preop.IV AP and oral post-op AP)	3285	No clear advantage of AP in reducing adverse effects; no benefit in prolonged AP; non difference in preop. and postop. regimens.	High clinical and statistical heterogeinity; high risk of bias of the included studies.
Forrester (SIS)	2020	Guidelines (Review)	AP in facial fractures	-	Use AP in peri-operative period for surgical fractures; avoid AP in non-surgical fractures; avoid pre-operative and post-operative AP.	Statements are expert opinion synthesis of evidence

**Table 5:** Summary of studies on antibiotic prophylaxis in burns (AP: antibiotic prophylaxis; AT: antibiotic therapy; RCT: randomized controlled trial; HLOS: hospital length of stay).

Author	Year	Study Type	Intervention	N. of Patients	Result	Limitations
Avni	2010	Systematic review and meta-analysis of RCTs	AP effect on reducing mortality	Not stated	Reduction in mortality and pneumonia rate with systemic prolonged AP; no effect with topical AP; increased antibiotic resistance rate.	Possible overlap of prolonged AP with AT; suspect of overuse of AP; results not valid for peri-operative AP.
Barajas-Nava	2014	Systematic review and meta-analysis of RCTs	AP efficacy on preventing wound infection, mortality, sepsis, HLOS	2117	No benefit of systemic and topical AP; increased risk of wound infection with silver sulfaziadine compared to dressing or skin substitutes; no benefit with perioperative AP.	Studies included at high risk of bias
Csenkey	2019	Systematic review and meta-analysis of RCTs and cohort	AP efficacy in preventing infective complications in pediatric burn injury (AP vs. noAP)	1735	No benefit from AP in local and systemic infective complications.	Reduced number of studies included (6 studies); mixed cohort studies and RCTs
Ramos	2017	Systematic review	Use of systemic AP in burn patients (adult and pediatric)	-	AP could be adequate in patients with severe burns requiring mechanical ventilation; perioperative AP could be useful in preventing split-thickness graft infection.	Wide time span of trail included (1982–2016); heterogeneity of type of AP, dose, and duration of AP.
Muthukumar	2019	Retrospective	Mortality and sepsis rate in burned patients treated with AP vs. noAP	157	No differences in mortality and sepsis; reduction of mortality of AP in patients with inhalation burns and pneumonia	Observational study, population size
Yeong	2020	Retrospective	Effect of AP on wound microbiology and outcomes in mass burn casualties	31	39% of patients developed multi-resistant pathogens; increased risk with >40% of body involved and with 2 or more antibiotic classes.	Observational study, population size, heterogeneity of AP regimens; mixed AP and AT

**Table 6:** Summary of studies on antibiotic prophylaxis in skin wounds and bites (AP: antibiotic prophylaxis; AT: antibiotic therapy; RCT: randomized controlled trial; ICU: intensive care unit).

Author	Year	Study Type	Intervention	N. of Patients	Result	Limitations
Gerhardt (Skin wounds)	2009	Retrospective	Effect on infection rate of wound irrigation and systemic AP in mild combat wounds	53	Probable benefit in synergic systemic AP + irrigation in reducing wound infection rate	Observational study, small sample size, mixed lesions
Lloyd (Skin wounds)	2018	Retrospective	Extended AP vs. narrow AP in combat-related soft tissues wounds	287	No difference between the two groups; no benefit in extended AP	Observational study, does not consider group with no AP as control.
Weintrob (Skin wounds)	2018	Prospective	Risk factors for early infection in combat injuries	1807	Antibiotic administration does not affect infection rate; infection rate is related to amputation, severity of injury, need of mechanical ventilation, ICU admission	Observational study, mixed kind of lesions, not clear discrimination between AP and AT
Medeiros (Bites)	2001 (updated 2009)	Systematic review and meta-analysis of RCTs	AP vs. noAP effect on Infection rate for mammalian bites	522	AP is effective in reducing infection rate in human bites; type of wound does not influence infection rate; hand bites have a higher rate of infection if not treated with AP	Inappropriate antibiotics, according to type of bacteria involved, were used in some studies
Looke (Bites)	2009	Systematic review	AP vs. noAP effect on infection rate in mammalian bites	-	AP has no effect in reducing infection rate in human or animal bites if risk areas (extremities and over cartilaginous areas) are not involved	Meta-analysis not performed; concerns about the quality of involved studies
